# Thymic Microenvironment: Interactions Between Innate Immune Cells and Developing Thymocytes

**DOI:** 10.3389/fimmu.2022.885280

**Published:** 2022-04-08

**Authors:** Helen Wang, Juan Carlos Zúñiga-Pflücker

**Affiliations:** ^1^ Department of Immunology, University of Toronto, Toronto, ON, Canada; ^2^ Biological Sciences, Sunnybrook Research Institute, Toronto, ON, Canada

**Keywords:** thymus, macrophage, dendritic cell, T cell development, positive selection, negative selection, thymus repair

## Abstract

The thymus is a crucial organ for the development of T cells. T cell progenitors first migrate from the bone marrow into the thymus. During the journey to become a mature T cell, progenitors require interactions with many different cell types within the thymic microenvironment, such as stromal cells, which include epithelial, mesenchymal and other non-T-lineage immune cells. There are two crucial decision steps that are required for generating mature T cells: positive and negative selection. Each of these two processes needs to be performed efficiently to produce functional MHC-restricted T cells, while simultaneously restricting the production of auto-reactive T cells. In each step, there are various cell types that are required for the process to be carried out suitably, such as scavengers to clean up apoptotic thymocytes that fail positive or negative selection, and antigen presenting cells to display self-antigens during positive and negative selection. In this review, we will focus on thymic non-T-lineage immune cells, particularly dendritic cells and macrophages, and the role they play in positive and negative selection. We will also examine recent advances in the understanding of their participation in thymus homeostasis and T cell development. This review will provide a perspective on how the thymic microenvironment contributes to thymocyte differentiation and T cell maturation.

## Introduction

The thymus is an essential organ for T cell development (1). It is home to many cell types, such as stromal and immune cells, which not only aid in T cell development, but are also integral to thymus homeostasis (2–4). During T cell development, bone marrow-derived early thymic progenitors (ETPs) first seed the thymus where they receive Notch signals from cortical thymic epithelial cells (cTECs) and are signaled to enter the T-lineage differentiation pathway (5). These early progenitor T cells are double negative (DN) for CD4 and CD8 expression and their T cell receptor (TCR) genes have not yet undergone V(D)J rearrangement (6). At this stage, DN cells rearrange their γ, δ and β TCR gene loci, and following successful TCRβ gene assembly gain CD4 and CD8 expressions, a checkpoint termed β-selection, and advance to the CD4 and CD8 double positive (DP) stage. Cells that properly rearrange their γδ TCRs mature into the γδ-T cell lineage (7). However, the majority of cells become DP cells, and following rearrangement of their TCRα gene loci are subjected to positive selection, which is conducted by cTECs presenting peptide self-antigens on their major histocompatibility complex (MHC) class I and MHC class II molecules to DP cells (8).

**Figure 1:**
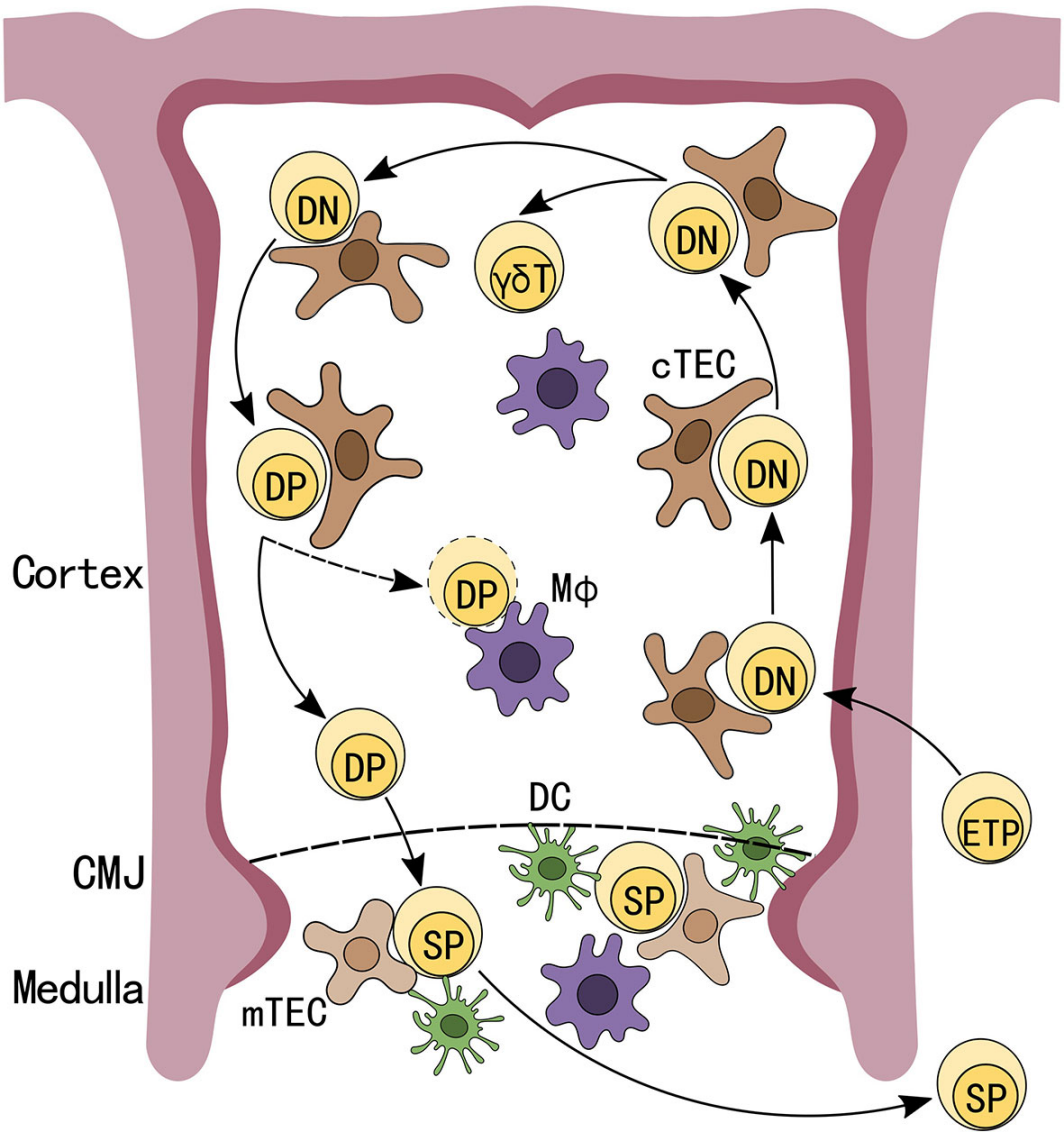
Schematic depiction of T cell development in the thymus. Early thymic progenitors (ETP), arriving from the bone marrow, seed the thymus and receive Notch signals from thymic epithelial cells (TECs) to differentiate into CD4^-^CD8^-^ double negative (DN) T-lineage cells. DN cells that have undergone successful V(D)J rearrangement at TCRβ gene loci differentiate into CD4^+^CD8^+^ double positive (DP) cells. After completing TCRα rearrangements and successfully undergoing positive selection, DPs migrate to the thymus medullary region and are subjected to negative selections while DPs that fail positive selection will be programmed for apoptosis. Cells that successfully passed these checkpoints will exit the thymus as CD4 or CD8 single positive (SP) T cells.

Proper TCR-MHC interactions predicate whether DP cells are allowed to differentiate to the next stage of αβ-T cell development. Conversely, DPs with non-functional TCR-MHC interactions undergo death by neglect, which occurs for over 95% of DPs (9, 10). Following positive selection, DPs migrate to the thymus medullary region and undergo negative selection against strong TCR-MHC interactions. This process, which helps to ensure self-tolerance, is conducted by medullary thymic epithelial cells (mTECs), which under the regulation of autoimmune regulator (AIRE) express a vast array of self-antigens, and with the help from other thymic antigen presenting cells (APC), such as dendritic cells (DCs) (**Figure 1**) (11–13). The purpose of this process is to eliminate potential self-reactive T cells, which could lead to autoimmune diseases if released into the periphery. In total, it is estimated that only 3-5% of developing thymocytes become mature CD4 or CD8 single positive (SP) T cells and exit the thymus (14).

The two-step selection process is repeated every day in the thymus and is only diminished during thymus aging or due to external injuries, such as irradiation and inflammatory stress (12, 15, 16). One necessary aspect of the selection process, which is critical to ensure that randomly generated TCRs are both able to properly interact with self-MHC and not lead to autoimmunity, is the need to eliminate a vast number of potentially useless or harmful cells on a continuous basis. Due to the daily massive cell death during T cell selection, thymic homeostasis needs to be strictly maintained by other cell types. Thymic macrophages are immune cells that are crucial for clearing apoptotic thymocytes in the thymus. Remarkably, thymic macrophages only make up 0.1% of all cells in the thymus (17). This suggests that they are highly efficient in efferocytosis since there are over 50 million DPs generated in a mouse thymus every day, a majority of which are likely destined for cell death and need to be cleared by thymic macrophages (13). These cells have also been shown to play a role in maintaining thymus homeostasis and thymus repair after injuries (18). As for the negative selection process, thymic DCs are also present in the medulla and have been shown to play a pivotal role in T cell selection alongside mTECs to curtail the generation self-reactive T cells and promote central tolerance (19). In this review, we will focus on these two important cell types in the thymus, DCs and macrophages, by examining their developmental origin, localization, function, and recent advances on their role in T cell selection and thymus repair post injury.

## Thymic Dendritic Cells

DCs in the thymus make up 0.5% of thymus cellularity and are mainly composed of three different groups: plasmacytoid DCs (pDCs), CD8^+^SIRPα^-^ (CD8^+^ DCs), and CD8^-^SIRPα^+^ (SIRPα^+^ DCs) (20). SIRPα^+^ DCs and pDCs are migratory DCs that developed in the bone marrow and migrate from the periphery to the thymus, while a small fraction of CD8^+^ DCs originate intrathymically from a common T/DC progenitor, majority of CD8^+^ DCs develop outside the thymus (21–26). Typically, mature SIRPα^+^ DCs are located in the cortico-medullary perivascular space, CD8^+^ DCs are located within the medulla, and pDCs are located at the cortical-medullary junction (CMJ) (27–29). A recent paper published by Sarah Teichman’s group using single-cell (sc) RNA-sequencing (seq) of human thymus cells, allowed them to identify a new subtype of DCs, which they named as activated DCs (aDCs), due to their high expression in costimulatory molecules (30). These aDCs could be further clustered into aDC1, aDC2, and aDC3 subsets, where aDC1 and aDC2 expressed similar gene profile as CD8^+^ DCs and SIRPα^+^ DCs, respectively. While the aDC3 cluster expressed lower levels of co-stimulatory molecules compared with other aDCs, suggesting that these are post-activated aDCs. The distinct gene expression profiles from the different aDCs subsets suggests they are derived from different DCs population. This new aDC subtype is located at the center of the medulla, and uniquely expresses *LAMP3* and *CCR7*, which are not found in other DC subtypes in the thymus. Their data also showed that aDCs can recruit naïve and regulatory T cells (Treg) into the thymus medullary through CCR7:CCL19 and CCR4:CCL17/CCL22 interactions, respectively. Interestingly, some aDCs also expressed AIRE, which validated other group’s previous findings (31, 32). It has been proposed that AIRE can regulate intercellular transfers of self-antigen from mTECs to thymic DCs to promote thymic tolerance (32, 33). Combined with their high costimulatory molecule expression and their interaction with developing T cells, these aDCs may play a role in T cell negative selection, however, functional analyses are needed to further determine the exact role that aDCs may play in T cell selection. Furthermore, whether these aDCs share a common developmental origin as CD8^+^ DCs and SIRPα^+^ DCs, or whether aDCs merely represent an activate stage of conventional DCs in the thymus requires further elucidation.

## Thymic Dendritic Cells on T Cell Selection

Thymic DCs are known to express high levels of class I and II MHC molecules (34). It has been well established that thymic DCs play a role in central tolerance and clonal deletion during T cell development (35). Particularly, SIRPα^+^ DCs have been shown to transport antigens through blood and induce Treg development in mice (36). Further validating this point, Dominik Filipp’s group recently found a novel CD14^+^SIRPα^+^ monocyte DCs (moDCs) subset in the thymus that was important for the generation of Tregs (37). While moDCs expressed some genes overlapping with SIRPα^+^ DCs, they also expressed high levels of monocyte associated genes (*Mafb*, *Apoe*, and *Csf2ra*), which are absent in the SIRPα^+^ DC subset, indicating that moDCs are likely a distinct population. Their findings suggested that the TLR9/MyD88 pathway induced mTECs to express chemokines that promoted the recruitment of moDCs to the thymus. These moDCs could also acquire antigens from mTECs. However, whether these or other DCs are able to transfer self-antigens expressed by medullary fibroblast, which were recently shown to express TRAs that contribute to central tolerance was not addressed (38, 39). Of note, MyD88^ΔTEC^ mice that conditionally lacked MyD88 in mTECs, there was a decrease in moDCs populations in the thymus, leading the impaired generation of Tregs, and those Tregs that were generated displayed reduced suppressive capacity. The same group also found specific DCs subsets in the thymus have a preference in antigen transfer from different TEC subsets (40). Notably, moDCs were most efficient in antigen transfer compared with all other thymic DC subsets, and moDCs were able to acquire antigens from multiple mTECs. However, the mechanism of how these cells acquire self-antigens for T cell negative selection remained unclear.

Attempting to answer the above questions, Charles J Kroger et al. illustrated how thymic DCs can acquire MHC molecules from TECs through intercellular transfer (41). By coculturing thymic DCs from NOD mice and TECs from BALB/c mice that express H2-D^d^ (an MHC class I antigen) and IE^d^ (an MHC class II antigen), the authors found thymic DCs, compared with splenic DCs, had a higher efficiency in acquiring H2-D^d^ and IE^d^. The capacity for MHC molecules uptake by thymus CD8^+^ DCs and SIRPα^+^ DCs were similar. However, this intercellular transfer ability was only found between thymic DCs and TECs, and not with other APCs, such as B cells, when cocultured with thymic DCs. Using qRT-PCR, the authors identified that this intercellular antigen transfer process was correlated with the unique expression of the epithelial marker EpCAM only in DCs found in the thymus. Thymic DCs were previously thought to acquire EpCAM protein from TECs, while this paper showed that both thymic CD8^+^ DCs and SIRPα^+^ DCs can express EpCAM, while SIRPα^+^ DCs expressed the highest level of EpCAM compared with all other DC subtypes in the thymus (42). This intercellular transfer ability in DCs is organ specific and is regulated differently between the different subsets of DCs in the thymus. This was shown when the authors blocked PI3K signaling and the transfer of MHC antigens to CD8^+^ DCs was reduced, while transfer to SIRPα^+^ DCs was not impacted. This work provided new insights on how thymic DCs can specifically acquire antigens from neighboring TECs in the thymus, and the mechanism for antigen transfer in thymus DCs subtypes are regulated by different pathways. Further studies can be done to determine the exact mechanism that regulates intercellular antigen transfer between TECs and SIRPα^+^ DCs in the thymus since these DCs are known to play a role in the generation of Tregs.

**Figure 2:**
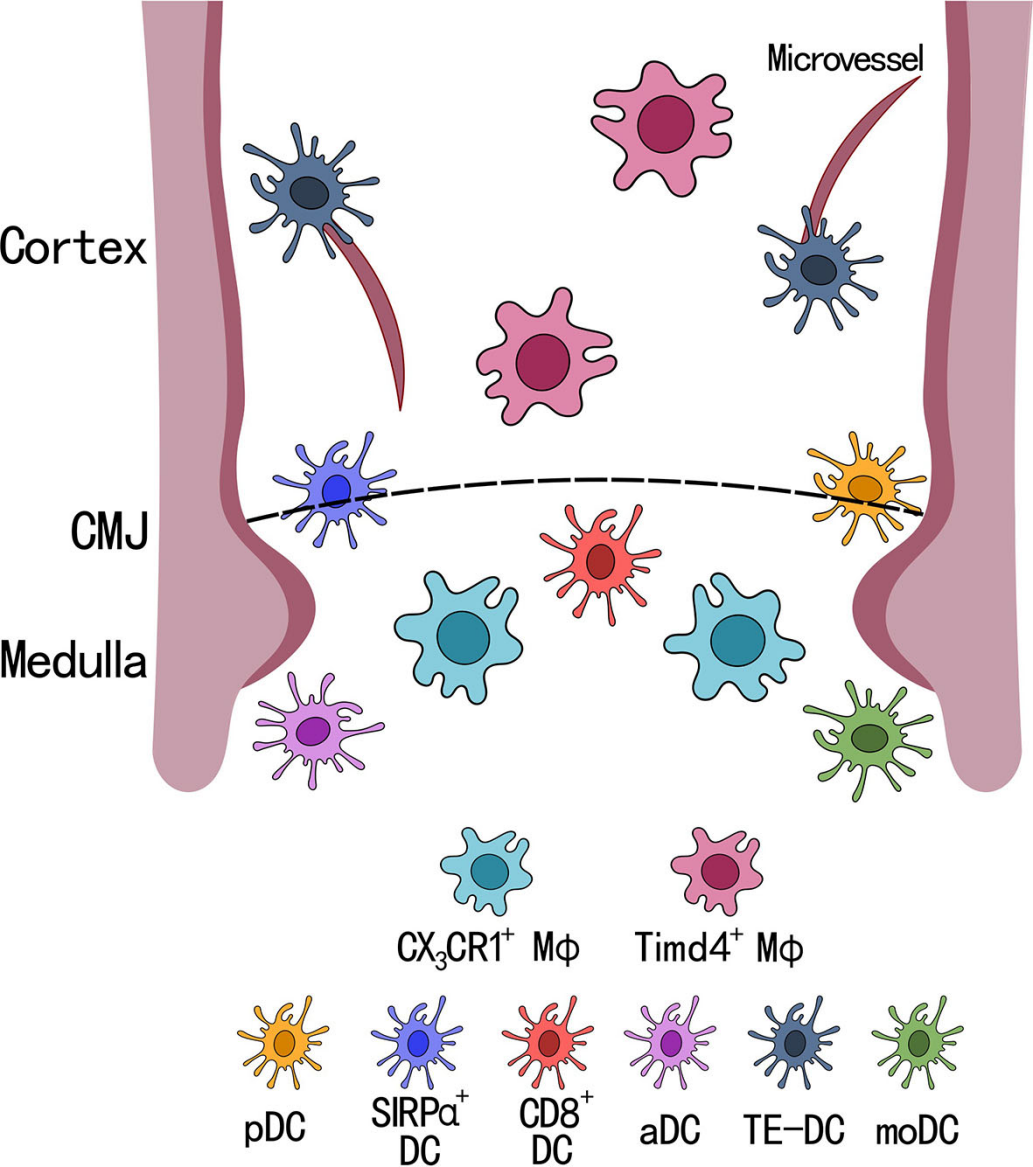
Localization of dendritic cell and macrophage subsets in the thymus. There are 6 subsets of dendritic cells (DCs) and 2 subsets of macrophage (MФ) in the thymus. SIRPα^+^ DCs and pDCs are located closely to the cortical-medullary junction (CMJ), CD8^+^ DCs, activated DCs (aDCs), and CD14^+^SIRPα^+^ moDCs (moDCs) are located within the medullary region, and transendothelial DCs (TE-DCs) are located between the microvessels in the thymus. Timd4^+^ macrophages are located within the cortex and uniquely express *Spic* and *Vcam1*, while CX_3_CR1^+^ macrophages are located at the CMJ expressing *Runx3* and antigen presenting genes, such as *H2-Q7*.

Because a majority of thymic DCs are periphery-derived that migrate to the thymus, they also have the capacity to carry antigens from the periphery to the thymus for T cell selection (35). However, the specific molecules that each thymic DCs subtype carries remains unclear. A recent paper from Ulrich von Adrian’s group found a new population of DCs that expresses CX_3_CR1 in both human and mice, which they named transendothelial DCs (TE-DCs) (43). Using multi-photon intravital microscopy, they found that these TE-DCs are located between the microvessels and the thymus where they can transport blood born proteins into the thymus and then use it for T cell selection (**Figure 2**). They also reported that these TE-DCs are a heterogeneous population of DCs, a majority of which are composed of SIRPα^+^ DCs, followed by pDCs. Only a small fraction of TE-DCs was identified as CD8^+^ DCs. This finding was supported by previous research that looked at the origin of thymic DCs and showed that SIRPα^+^ DCs and pDCs were migratory DCs from the periphery, while CD8^+^ DCs can be intrathymically derived. This new antigen transport system by CX_3_CR1 TE-DCs depends on its ligand CX_3_CL1, which is expressed by thymus endothelial cells. Recent work by Gretchen Diehl’s group also showed CX_3_CR1^+^ DCs can capture microbial antigens, present these antigens to developing T cells, and induce microbial-specific T cell expansion (44). Altogether, these findings introduced a new model for T cell selection by thymic DCs where a specialized subset of CX_3_CR1^+^ DCs, located at microvessels, are actively taking up blood born antigens and transporting them into the thymus for T cell selection. However, whether these CX_3_CR1^+^ DCs have distinct developmental origin and what signals are responsible for the polarization of CX_3_CR1 DCs are still unclear.

## Thymic Dendritic Cells in Post Infection

It has been shown that the generation of mature T cells from the thymus is attenuated during and post infections (45, 46). Since a majority of thymic DCs come from the periphery, whether migratory DCs play a role in thymus damage post infection was unclear. A recent publication by Haojie Wu et al. showed that mature DCs from the circulation can enter the thymus and induce thymus involution through the Notch signaling pathway (47). Upon activation by antigens such as lipopolysaccharide and ovalbumin, DCs have been shown to enhance Jagged1 expression (48, 49). Their work showed that these activated DCs expressing Jagged1 can bind to Notch3-expressing mTECs and this interaction through the Notch signaling pathway induces apoptosis in mTECs. This in turn led to the disruption in SP cell generation in the thymus. However, this finding needs to be validated in disease models, such as post viral infections. Nonetheless, this work provided a new perspective on thymic atrophy upon infection by activated DCs, suggesting that DCs in the thymus may play a deleterious role during an infection, which as previously thought that this may be critical to prevent the thymus from inducing self-tolerance against virally encoded antigens. It would also be interesting to test whether blocking DCs infiltration into the thymus post infection could prevent thymic atrophy.

## Thymic Macrophages

During T cell development, cells that do not pass positive or negative selection undergo apoptosis (50). It is estimated that over 95% of cells undergo apoptosis in the thymus every day (50, 51). However, when isolating cells from the thymus of healthy adult mice, one typically finds that nearly all the thymocytes are live cells, suggesting that apoptotic cells within the thymus are actively and effectively cleared (52, 53). The clearing of apoptotic cells is done by intrathymic macrophages (9, 30, 50, 54–56). For many years, macrophages in the thymus have not been well characterized nor understood, due to technical limitations in analyzing these cells and performing functional studies. There are only a few well known macrophage markers that have been found to be expressed on thymic macrophages (ED1 and ED2 in rats, CD68, F4/80 and CD11b in mice) making it difficult to study the origin of these thymic macrophages and identify their heterogeneity in the thymus (57–60). With the advent of scRNA-seq technology, characterizing small cell populations, and performing ontogeny analysis on thymic macrophages have become possible.

A recent publication by Tyng-An Zhou et al. identified two macrophage subsets (Timd4^+^ and CX_3_CR1^+^) in the thymus of adult mice using scRNA-seq (**Figure 2**). Both populations of thymic macrophages were found to developed during embryonic life, and the authors found Timd4^+^ thymic macrophages were derived from CX_3_CR1^+^ cells during embryogenesis. The two different subsets of thymic macrophages showed distinct gene expression profiles, where Timd4^+^ thymic macrophages expressed high levels of *SpiC*, *MafB*, and *Vcam1*, which showed high similarity with the transcriptomic landscape of spleen red pulp macrophages (61, 62). While CX_3_CR1^+^ thymic macrophages had high expression of *Runx3* (which is important for cytotoxic CD8^+^ T cell development), and genes involved in antigen presentation (*B2m*, *H2-M2, H2-K1, and H2-Q7*) (63–66). These two tissue resident macrophage subsets found in the thymus agreed with recent findings by Slava Epelman’s group, in which they showed Timd4^+^ and CX_3_CR1^+^ tissue resident macrophages were found across many organs (heart, liver, lung, kidney, and brain) in mice (67).

The distinct gene profile for these two subsets of thymic macrophages suggested they may have different functions within the thymus. Using immunofluorescence to examine thymic histological sections, Zhou et al. found that Timd4^+^ macrophages are found mainly in the cortex, while CX_3_CR1^+^ macrophages are localized in the CMJ. In combination with their transcriptomic profile, this suggests that Timd4^+^ thymic macrophages are the main cells performing efferocytosis of apoptotic thymocytes. Their findings were also supported by Catherine C. Hedrick’s group who demonstrated that Timd4^+^F4/80^+^ thymic macrophages have the highest phagocytic efficiency compared with other macrophage subsets, and that the depletion of these macrophages accelerated thymic involution, suggesting an important role in thymic homeostasis (68).

Conversely, CX_3_CR1^+^ thymic macrophages may play a role in T cell negative selection. This is supported by their location at the CMJ, which is where negative selection initiates, as positively selected thymocytes migrate into the medulla. Combined with their gene expression profile and migratory ability, these thymic macrophages may have the potential to carry self-antigen through blood vessels and present them to developing T cells for negative selection and tolerance induction. However, further studies need to be performed to validate their potential functions *in vivo* (69).

## Thymic Macrophage in T Cell Selection

As the findings from Zhou et al. suggest, thymic macrophages may play a role in T cell selection by their antigen presenting ability. Other groups have shown Timd4^+^ cells in the thymus can also present MHC-I peptides and induce negative selection of CD8^+^ T cells (70, 71). However, as these authors mentioned, Timd4 can also be expressed on thymic DCs, thus it is difficult to distinguish whether Timd4^+^ thymic macrophage are the true players for culling self-reactive CD8^+^ T cells and whether they play a defining role in presenting antigens to developing T cells during negative selection. These data contrast the scRNA-seq results presented by Zhou’s group, where CX_3_CR1^+^ thymic macrophages by their location and gene expression profile were suggested to have a higher probability in presenting self-antigens for negative selection.

Vijay K. Kuchroo’s group generated a Timd4^-/-^ mice, and found that Timd4-deficient mice had hyperactive T and B cells, as well as displaying an impairment in efferocytosis by peritoneal macrophages (70). However, the absolute cell number of thymocytes in Timd4 deficient mice did not differ from control wild-type mice, which contrasts other group’s findings, where the depletion of thymic macrophage led to an acceleration of thymic involution, and hence decreasing the size of the thymus (68, 71). This could be attributed by the compensation from other phagocytes in the thymus of Timd4^-/-^ mice to maintain thymus homeostasis. This was evidenced in other organs where depletion of a specific subset of tissue resident macrophages led to empty niches in the organ where infiltrating monocytes or other tissue resident macrophages quickly occupied these niches and performed functions similar to the original tissue resident macrophage (72–74). Thus, whether thymic macrophages play a role in T cell selection remains to be elucidated.

## Thymic Macrophage During Thymus Injury

In addition to efferocytosis, phagocytosis and antigen presentation, tissue resident macrophages have been shown to play a crucial role in tissue repair across many organs (75–77). After tissue injury, tissue resident macrophages can secrete cytokines (IL-10 and TGFβ), growth factors (FGF, TGFα, and PDGF), and exosomes to promote cell differentiation and suppress inflammation (78). Depletion of tissue resident macrophages in the heart and liver were shown to impair organ healing (67, 76, 79–81). However, whether thymic macrophages can play a similar role in thymus repair is still unclear.

One clinically relevant source of injury to the thymus is irradiation, a process that some cancer patients are subjected to as part of their treatment (82, 83). The rate of recovery is crucial as the thymus is integral for generating T cells that form an immune response. Several groups have sought new approaches to improve thymic recovery post irradiation treatment (84–86). A recent publication by Gen Yamada’s group used a *MafB*/green fluorescent protein knock-in (*MafB* ^+/GFP^) mouse to demonstrated that MafB expressing cells in the thymus play a crucial role in thymus repair after irradiation. When comparing thymus recovery post irradiation between *MafB* ^+/+^ and *MafB* ^+/GFP^, the authors found that there was a decrease in immature TECs (Krt5^+^FoxN1^+^) generated in the *MafB* ^+/GFP^ thymus. The organization of the medulla was also found to be abnormal post-irradiation injury, where mTECs in the *MafB* ^+/GFP^ thymus formed only one prominent medullary compartment, while *MafB* ^+/+^ maintained multiple medullary compartments after recovery. Since *MafB* is a common marker used to identify macrophage populations, it stands to reason that a majority of the cells expressing *MafB* in the thymus are likely macrophages (17, 18, 87). This new finding showed that thymic macrophages may play a role in thymus repair, potentially by engulfing apoptotic cells and controlling inflammation in the thymus. These results also suggested that post thymic injury, macrophages are important for the repair of the thymus architecture and supporting the regeneration of thymic endothelial cells. However, exactly which of the two thymic macrophage populations is playing a role in thymus repair after injury remains unclear. Further studies are needed to assess the role of the two thymic macrophage subsets, Timd4^+^ and CX_3_CR1^+^, in clinically relevant injury models.

## Conclusion

The thymus is a sophisticated organ that is important for generating T cells, which play a critical role in immune function. As a result, severe consequences can arise if thymic homeostasis is not properly regulated. This therefore demands the need to have a thorough understanding of the thymus environment that induces and support T cell development. Although the T cell selection process by TECs has been well studied, whether thymic DCs and macrophages are important players in T cell development, selection and thymus homeostasis remain to be further elucidated. With scRNA-seq technology, several groups have been able to identify new populations of DCs in the thymus (aDCs, TE-DCs, and CX_3_CR1^+^ DCs), each of which appears to serve distinct functions. Macrophage heterogeneity in the thymus was also elucidated using this technology, and we can now appreciate that there are two macrophage populations in the thymus, Timd4^+^ and CX_3_CR1^+^. However, there are still many questions remaining, such as which thymic macrophage subset plays a role in thymus repair? Do thymic macrophages play a role in the negative selection of T cells, if so, which subset? By addressing these questions, we can pave the way to promoting new clinical therapies for the repairing of the thymus post injuries.

## Author Contributions

HW wrote the manuscript. JCZ-P wrote and edited the manuscript. All authors contributed to the article and approved the submitted version.

## Funding

This work was supported by a grant from the Canadian Institutes of Health Research (CHIR, FND-154332).

## Conflict of Interest

The authors declare that the research was conducted in the absence of any commercial or financial relationships that could be construed as a potential conflict of interest.

## Publisher’s Note

All claims expressed in this article are solely those of the authors and do not necessarily represent those of their affiliated organizations, or those of the publisher, the editors and the reviewers. Any product that may be evaluated in this article, or claim that may be made by its manufacturer, is not guaranteed or endorsed by the publisher.
